# The Effect of Australian and Asian Commercial Antivenoms in Reversing the Post-Synaptic Neurotoxicity of *O. hannah*, *N. naja* and *N. kaouthia* Venoms In Vitro

**DOI:** 10.3390/toxins14040277

**Published:** 2022-04-13

**Authors:** Tam M. Huynh, Wayne C. Hodgson, Geoffrey K. Isbister, Anjana Silva

**Affiliations:** 1 Monash Venom Group, Department of Pharmacology, Biomedical Discovery Institute, Monash University, Clayton, VIC 3800, Australia; tommy.huynh@monash.edu (T.M.H.); geoff.isbister@gmail.com (G.K.I.); nkanjanasilva@gmail.com (A.S.); 2 Clinical Toxicology Research Group, University of Newcastle, Newcastle, NSW 2298, Australia; 3 Department of Parasitology, Faculty of Medicine and Allied Sciences, Rajarata University of Sri Lanka, Anuradhapura 50008, Sri Lanka

**Keywords:** neurotoxin, postsynaptic, snake, antivenom, neuromuscular, skeletal muscle

## Abstract

Despite antivenoms being the only established specific treatment for neuromuscular paralysis arising from snake envenoming, their ability to reverse the post-synaptic neurotoxicity in snake envenoming is poorly understood. We investigated the ability of five commercial antivenoms i.e., King cobra monovalent, Thai cobra monovalent, Thai neuro polyvalent, Indian polyvalent and Australian polyvalent antivenoms to reverse neurotoxicity induced by the venoms of King cobra (*Ophiophagus hannah*, 3 µg/mL), Indian cobra *(Naja naja*, 5 µg/mL) and Thai cobra (*Naja kaouthia*, 3 µg/mL) using the in vitro chick-biventer cervicis nerve–muscle preparation. All three venoms displayed post-synaptic neurotoxicity, which was prevented by all tested antivenoms (40 µL/mL) added to the bath prior to venom. All antivenoms partially reversed the established post-synaptic neuromuscular block after the addition of the three venoms during a 180 min observation period, but to varying degrees and at different rates. The neurotoxic effects of *O. hannah* venom recovered to a greater magnitude (based on twitch height restoration) and faster than the neurotoxicity of *N. kaouthia* venom, which recovered to a lower magnitude more slowly. The recovery of post-synaptic neurotoxicity by *N. naja* venom was hindered due to the likely presence of cytotoxins in the venom, which cause direct muscle damage. The observations made in this study provide further evidence that the commercial antivenoms are likely to actively reverse established α-neurotoxin-mediated neuromuscular paralysis in snake envenoming, and there is cross-neutralisation with different antivenoms.

## 1. Introduction

Snakebites are a global health concern that frequently causes illness and death in rural tropics, where access to prompt specific treatment and clinical reporting are poor [1]. A common clinical effect of elapid envenoming, and envenoming by some viperid snakes, is neuromuscular paralysis. This manifests in a range of severity, from mild paralysis, limited to facial muscle weaknesses, to life-threatening respiratory and limb paralysis in the most severe cases [2]. Snake venom neurotoxins cause paralysis by blocking neurotransmission in skeletal muscles and either act pre-synaptically (β-neurotoxins) or post-synaptically (α-neurotoxins) at the neuromuscular junction. α-Neurotoxins block neurotransmission by binding with the two acetylcholine binding sites on the nicotinic acetylcholine receptor (nAChRs) on the motor-end plate [3] and are further classified as either short-chain or long-chain α-neurotoxins based on their structural and functional differences [3,4]. Long-chain α-neurotoxins are likely to be more clinically relevant in human neuromuscular paralysis due to being more potent and less reversible at human nAChRs [4]. 

The combined use of antivenom with supportive treatment, e.g., mechanical ventilation, is required for treating victims with life-threatening neurotoxic envenoming [2]. The role of antivenom in the treatment of snakebite paralysis is to “trap” the neurotoxins in the circulation and at the neuromuscular junction [2,5], to prevent the neurotoxins binding to the AChR. However, the role of antivenoms in the reversal of α-neurotoxin-mediated neuromuscular paralysis remains unclear, i.e., the ability of antivenom to neutralize the effects of the α-neurotoxins once they are bound to the AChRs. Commercial antivenoms and monoclonal antibodies have been shown to accelerate the dissociation of short-chain α-neurotoxins from the nicotinic acetylcholine receptor and toxin complex [4]. However, a number of studies have shown that long-chain neurotoxins are only partially reversible, while some show greater reversibility [4,6]. In contrast, neuromuscular paralysis caused by pre-synaptic neurotoxins is considered to be irreversible and, therefore, resistant to antivenom therapy, as evident from both experimental and clinical studies. This is due to the irreversible nature of the motor-nerve terminal damage [2,5,7]. Clinical reports of neurotoxic envenoming by snake species with venoms containing predominantly α-neurotoxins, such as the Thai cobra (*Naja kaouthia*), have shown patients recovering with antivenom [8]. Hence, the efficacy of antivenom treatment against post-synaptic neurotoxic snake venoms needs further investigation. 

Here, we aim to investigate the ability of three commercial antivenoms to reverse the in vitro neurotoxicity induced by the venom from three different Asian cobra species, King cobra (*O. hannah*), Indian cobra (*N. naja*) and Thai cobra (*N. kaouthia*), which contain predominantly α-neurotoxins and lack pre-synaptic neurotoxins [9,10,11,12,13,14]. 

## 2. Results

### 2.1. Prevention of In Vitro Neurotoxicity due to O. hannah, N. kaouthia and N. naja Venoms with Antivenom

**Figure 1:**
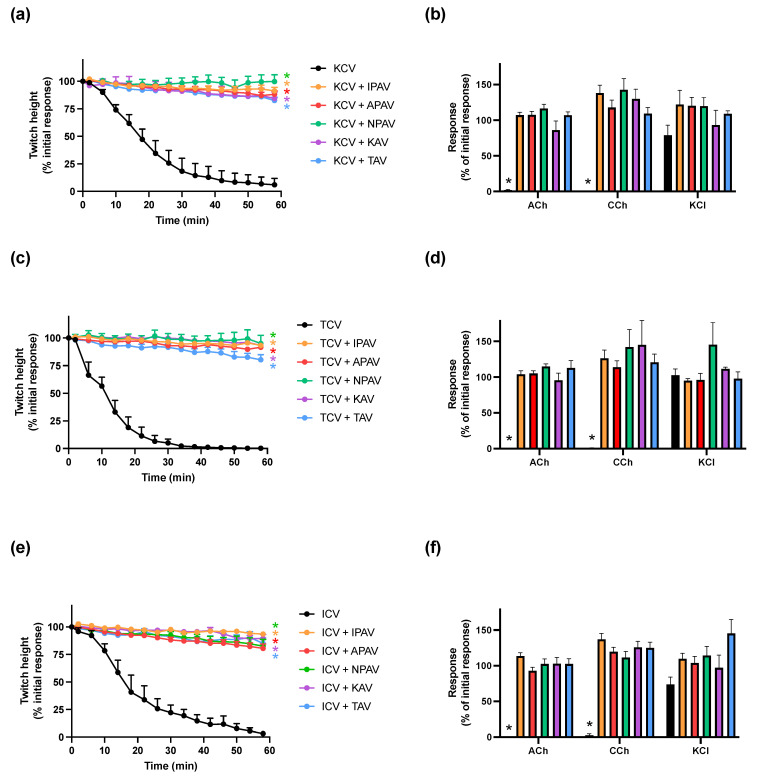
Cross-neutralization of the neurotoxicity of *O. hannah* (KCV; 3 µg/mL), *N. kaouthia* (TCV; 3 µg/mL) and *N. naja* (ICV; 5 µg/mL) venoms by King cobra antivenom (KAV), Thai cobra antivenom (TAV), Thai neuro polyvalent antivenom (NPAV), Indian polyvalent antivenom (IPAV) or Australian polyvalent antivenom (APAV): Panels (**a**,**c**,**e**) show the prevention of the inhibition of indirect twitches by *O. hannah*, *N. kaouthia* and *N. naja* venoms, respectively (* *p* < 0.05, significantly different from venom at 60 min, one-way ANOVA followed by Bonferroni’s post hoc test; *n* = 5); Panels (**b**,**d**,**f**) show the effect of *O. hannah*, *N. kaouthia* and *N. naja* venoms, respectively, on contractile responses to exogenous agonists (ACh; 1 mM, CCh; 20 µM and KCl; 40 mM) in the absence and presence of antivenoms (* *p* < 0.05, significantly different from the response to the same agonist before addition of venom, paired *t* test; *n* = 5; error bars indicate standard error of the mean).

*O. hannah* (3 µg/mL), *N. kaouthia* (3 µg/mL) and *N. naja* (5 µg/mL) venoms inhibited indirect twitches of the chick biventer nerve–muscle preparation (*n* = 5; *p* < 0.05, one-way ANOVA; Figure 1a,c,e). All three venoms also abolished contractile responses to exogenous ACh and CCh, but not KCl, indicating post-synaptic activity at the neuromuscular junction (*n* = 5; *p* < 0.05, paired *t* test; Figure 1b,d,f). The times taken to cause 90% inhibition of the indirect twitches (i.e., *t_90_*) following *O. hannah*, *N. kaouthia* and *N. naja* venom addition, compared to pre-venom twitch height were 42, 23 and 42 min, respectively.

All five antivenoms (40 µL/mL) prevented the reduction of indirect twitches by *O. hannah*, *N. kaouthia* and *N. naja* venoms (*n* = 5; *p* < 0.05, one-way ANOVA; Figure 1a,c,e), and prevented the inhibition of contractile responses to ACh and CCh (*n* = 5; *p* < 0.05, paired *t* test; Figure 1b,d,f), when added 10 min prior to the venoms.

### 2.2. Reversal of In Vitro Neurotoxicity of Venoms by Different Antivenoms

#### 2.2.1. Reversal of In Vitro Neurotoxicity by *O. hannah* Venom by Antivenoms

**Figure 2:**
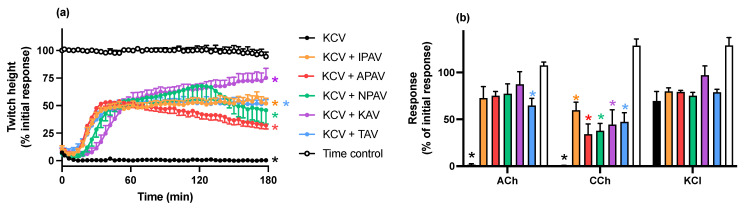
The effect of the addition of King cobra (KAV), Thai cobra (TAV), Thai neuro polyvalent (NPAV), Indian polyvalent (IPAV) or Australian polyvalent (APAV) antivenoms, added at the *t_90_* time point (time = 0 min), on the neurotoxicity of *O. hannah* venom (KCV; 3 µg/mL). Panel (**a**) shows the partial reversal of the inhibition of indirect twitches (* *p* < 0.05, significantly different from time control at 180 min, one-way ANOVA followed by Bonferroni’s post hoc test; *n* = 4–5). Panel (**b**) shows the effect of *O. hannah* venom on responses to exogenous agonists (ACh; 1 mM, CCh; 20 µM and KCl; 40 mM) in the absence and presence of antivenoms (* *p* < 0.05, significantly different from pre-toxin response to same agonist, paired *t* test; *n* = 4–5; error bars indicate standard error of the mean).

The addition of each antivenom (40 µL/mL), at the *t_90_* time point following the addition of *O. hannah* venom (3 µg/mL), partially restored the indirect twitches by the 180 min time point, although twitch height was not fully restored compared to control (*n* = 4–5; *p* < 0.05, one-way ANOVA; Figure 2a). The magnitude of the reversal was greatest for the specific King cobra antivenom with the Australian antivenom being least effective. The venom-induced reduction of contractile responses to ACh and CCh were also partially reversed by each of the antivenoms (*n* = 4–5; *p* < 0.05, paired *t* test; Figure 2b).

#### 2.2.2. Reversal of In Vitro Neurotoxicity by *N. kaouthia* Venom by Antivenoms

**Figure 3:**
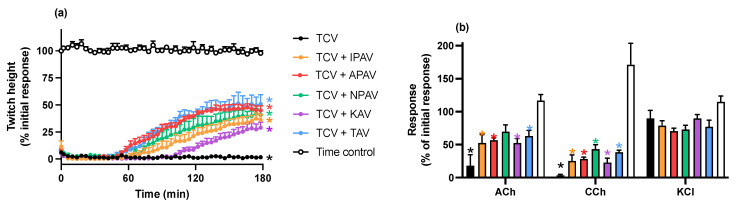
The effect of the addition of King cobra (KAV), Thai cobra (TAV), Thai neuro polyvalent (NPAV), Indian polyvalent (IPAV) or Australian polyvalent (APAV) antivenoms, added at the *t_90_* time point (time = 0 min), on the neurotoxicity of *N. kaouthia* venom (TCV; 3 µg/mL). Panel (**a**) shows the partial reversal of the inhibition of indirect twitches (* *p* < 0.05, significantly different from time control at 180 min, one-way ANOVA followed by Bonferroni’s post hoc test; *n* = 4–5). Panel (**b**) shows the effect of *N. kaouthia* venom on responses to exogenous agonists (ACh; 1 mM, CCh; 20 µM and KCl; 40 mM) in the absence and presence of antivenoms (* *p* < 0.05, significantly different from pre-toxin response to same agonist, paired *t* test; *n* = 4–5; error bars indicate standard error of the mean).

The addition of each antivenom (40 µL/mL), at the *t_90_* time point following the addition of *N. kaouthia* venom (3 µg/mL), partially restored the indirect twitches by the 180 min time point, although twitch height was not fully restored compared to control (*n* = 4–5; *p* < 0.05, one-way ANOVA; Figure 3a). The magnitude of the reversal was greatest for the specific Thai cobra antivenom, with the King cobra antivenom being least effective. The venom-induced reduction of contractile responses to ACh and CCh were also partially reversed by each of the antivenoms (*n* = 4–5; *p* < 0.05, paired *t* test; Figure 3b).

#### 2.2.3. Reversal of In Vitro Neurotoxicity by *N. naja* Venom by Antivenoms

**Figure 4:**
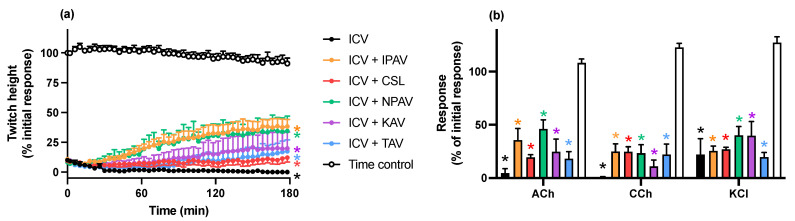
The effect of addition of King cobra (KAV), Thai cobra (TAV), Thai neuro polyvalent (NPAV), Indian polyvalent (IPAV) or Australian polyvalent (APAV) antivenoms, added at the *t_90_* time point (time = 0 min), on the neurotoxicity of *N. naja* (ICV; 5 µg/mL) venom. Panel (**a**) shows the partial reversal of the inhibition of indirect twitches (* *p* < 0.05, significantly different from time control at 180 min, one-way ANOVA followed by Bonferroni’s post hoc test; *n* = 4–5). Panel (**b**) shows the effect of *N. naja* venom on responses to exogenous agonists (ACh; 1 mM, CCh; 20 µM and KCl; 40 mM) in the absence and presence of antivenoms (* *p* < 0.05, significantly different from pre-toxin response to same agonist, paired *t* test; *n* = 4–5; error bars indicate standard error of the mean).

The addition of each antivenom (40 µL/mL), at the *t_90_* time point following the addition of *N. naja* venom (3 µg/mL), partially restored the indirect twitches by the 180 min time point, although twitch height was not fully restored compared to control (*n* = 4–5; *p* < 0.05, one-way ANOVA; Figure 4a) of the pre-venom twitch height at 180 min. The magnitude of the reversal was greatest for the specific Indian cobra antivenom, with the Australian antivenom being least effective. The venom-induced reduction of contractile responses to ACh and CCh were partially reversed by each of the antivenoms (*n* = 4–5; *p* < 0.05, paired *t* test; Figure 4b). The reduction of responses to KCl caused by the venom was not reversed by antivenoms.

## 3. Discussion

We have shown that the in vitro neurotoxicity of *O. hannah*, *N. kaouthia and N. naja* venoms can be fully prevented by all tested antivenoms when the antivenoms are added prior to venom addition, indicating efficacy against the neurotoxins in these venoms. When added after the venoms, the antivenoms partially reversed the established post-synaptic neuromuscular block by the three venoms during the 180 min observation period, but at varying degrees and rates, with the effect of *O. hannah* venom recovering greater and faster compared to *N. kaouthia* venom, which showed a comparatively lesser and delayed recovery to all antivenoms. In each case, the most efficacious antivenom, as indicated by the magnitude of recovery) was the specific antivenom raised against the venom of the species being studied. The recovery of the post-synaptic neurotoxicity by *N. naja* venom was hindered (i.e., displayed the lowest magnitude of recovery) due to the presence of cytotoxins in this venom which cause direct muscle damage [15]. 

*O. hannah*, *N. kaouthia* and *N. naja* venoms all displayed post-synaptic neurotoxicity as indicated by the in vitro inhibition of indirect twitches and contractile responses to ACh and CCh, but not KCl. This is consistent with proteomic studies which suggest the dominance of α-neurotoxins in these venoms [9,10,11,16,17,18,19,20]. The post-synaptic neurotoxicity of all venoms was neutralised by all five tested Asian and Australian antivenoms. Additionally, the effective cross-neutralisation of the in vitro neurotoxicity of these venoms by heterologous antivenoms was demonstrated, which is likely due to the α-neurotoxins in these venoms sharing common antigenic regions. The in vitro cross-neutralisation of neurotoxic snake venom by antivenoms raised against different snakes has been demonstrated previously [21,22,23]. However, although these observations would not be readily translated to the clinical effectiveness of antivenoms since, in our studies, antivenom was pre-incubated in the organ bath. 

To examine the ability of antivenom to reverse established in vitro neurotoxicity, we performed antivenom reversal studies by adding antivenom at the *t_90_* time point. All antivenoms partially reversed the post-synaptic neurotoxicity of both *O. hannah* and *N. kaouthia* venoms. Since both venoms contain mostly α-neurotoxins [10,11], the observed in vitro neurotoxicity could be entirely attributed to α-neurotoxins. Hence, the partial reversal of these venoms may suggest the presence of both reversible and pseudo-irreversible α-neurotoxins. The recovery of tissues from *O. hannah* venom-induced neurotoxicity occurred noticeably earlier, reaching approximately 50% of initial twitch height by 60 min after antivenom was added. In contrast, the reversal of *N. kaouthia* venom-induced neurotoxicity only recovered by approximately 25–50% after 180 min. The differences in reversibility between *O. hannah* and *N. kaouthia* venoms may be due to the greater abundance of long-chain α-neurotoxins in *N. kaouthia* venom [10,11], since long-chain α-neurotoxins are typically less reversible compared to short-chain α-neurotoxins [4]. 

The neurotoxic effects of *N. naja* venom were less reversible by antivenom compared to those induced by *O. hannah* or *N. kaouthia* venoms. By 180 min after antivenom administration, the antivenoms partially restored the twitches to 10–35% of initial twitch height. Additionally, *N. naja* venom also caused a significant reduction in contractile responses to KCl, which could not be reversed by the addition of antivenoms. It is likely that direct muscle damage caused by *N. naja* venom has occurred in these experiments rather than tissue fatigue [7], since a significant decrease in KCl responses was not observed in antivenom reversal studies for *O. hannah* and *N. kaouthia* venoms. The presence of three-finger cytotoxins, called cardiotoxins, has been identified in *N. naja* venom by proteomic studies [24,25]. It is possible that long-chain α-neurotoxins contribute to the lack of reversibility observed in antivenom reversal studies for *N. naja* venom, but the cardiotoxins likely play a role in causing irreversible muscle damage, which is evident by the reduction in KCl responses.

The observations made in this study provide further evidence that the role of antivenom in treating post-synaptic neurotoxicity in snake envenoming is beyond the mere trapping of toxins within circulation and static hindrance. More importantly, our experiments showed that antibodies, from both specific and non-specific antivenoms, are likely to be disrupting the α-neurotoxin-nAChR interaction in an active manner, suggesting that the antivenoms are likely to reverse the α-neurotoxin-mediated established neuromuscular paralysis in snake envenoming. Previously, it has been observed that the dissociation of toxin-α, a short-chain α-neurotoxin from *Naja nigricollis* venom, from the nAChR could be markedly accelerated by homologous monoclonal and polyclonal antibodies [26,27,28]. Further experiments using animal models may shed further light on these interactions, although we have recently cautioned about the interpretation of results using rodent lethality models when evaluating antivenoms [29]. The conclusions drawn from our experiments are subject to some limitations. First, the quantity of homologous and heterologous antivenoms (40 µL/mL) used in the current study was higher than the manufacturer-recommended amounts of the respective antivenoms. The quantity of antivenom used was based on our intention to ensure the presence of high amounts of anti-α-neurotoxin antibodies in order to neutralise the post-synaptic neurotoxicity as a “proof of concept”.

In addition, in our study, the venom has more direct access to the tissue than would be experienced in a clinical situation. Therefore, the model we used does not represent the pharmacokinetics of the toxins or antivenoms in human envenoming but only reflects the pharmacodynamic interactions. Hence, in our studies, the onset of action of the venom/toxins will be accelerated, and the antivenom is likely to have more rapid and greater access to the venom than would occur clinically.

## 4. Conclusions

We demonstrated that the in vitro post-synaptic neurotoxicity of *O. hannah*, *N. kaouthia* and *N. naja* venoms can be cross-neutralised and partially reversed by five Asian and Australian antivenoms. The neurotoxic effects of *N. kaouthia* venom were less reversible than *O. hannah* venom, possibly due to *N. kaouthia* venom containing more irreversible α-neurotoxins. The even lower reversibility displayed by *N. naja* venom is likely to be attributed to the cytotoxic effects of the venom. The observations made in this study suggest that commercial antivenoms have the capacity to actively reverse established α-neurotoxin-mediated neuromuscular paralysis in snakebite envenoming. However, further testing in other is warranted to better understand the true potential of antivenom therapeutics in the setting of α-neurotoxin-dominant snakebite envenoming syndromes such as paralysis.

## 5. Materials and Methods

### 5.1. Venoms and Antivenoms

We used the following venoms: *N. kaouthia* venom and Indonesian *O. hannah* venom from Venom Supplies (Tanunda, South Australia, Australia), and *N. naja* venom from Sri Lanka. Freeze-dried venoms were dissolved in 0.05% (*w*/*v*) bovine serum albumin and stored at −20 °C until required. We used the following antivenoms: King cobra monovalent antivenom (KCAV; Thai Red Cross Society; Bangkok, Thailand; Batch No: LH00118, expiry date: 13/02/2023), Thai cobra monovalent antivenom (TCAV; Thai Red Cross Society; Bangkok, Thailand; Batch No: NK00316, expiry date: 11/10/2021), Thai neuro polyvalent antivenom (NPAV; raised against *Bungarus candidus*, *B. fasciatus*, *O. hannah*, and *N. kaouthia*; Thai Red Cross Society; Bangkok, Thailand; Batch No: NP00120, expiry date: 27/03/2025), Indian polyvalent antivenom (IPAV; raised against *Bungarus caeruleus*, *Naja*, *Daboia russelii* and *Echis carinatus*; VINS Bioproducts, Telangana, India; Batch no: 01AS11005, expiry date: 13/02/2023) and Australian polyvalent antivenom (APAV; raised against *Pseudechis australis*, *Notechis scutatus*, *Pseudonaja textillis*, *Acanthophis antarticus* and *Oxyuranus scutellatus*; CSL; Parkville, Australia; Batch No: 055517501; expiry date: 04/2014). For all experiments, antivenoms were tested at 40 µL/mL bath concentrations to achieve a sufficiently high concentration of antivenom for the venom.

### 5.2. Chemical and Reagents

The following consumables were used for experiments: acetylcholine chloride (ACh; Sigma-Aldrich, St. Louis, MO, USA), carbamylcholine chloride (CCh; Sigma-Aldrich, St. Louis, MO, USA), potassium chloride (KCl; Ajax Finechem Pty. Ltd., Taren Point, Australia), d-tubocurarine (Sigma-Aldrich, St. Louis, MO, USA) and bovine serum albumin (BSA; Sigma-Aldrich, St. Louis, MO, USA). All chemicals were dissolved or diluted in milli-Q water.

### 5.3. Chick Biventer Cervicis Nerve-Muscle Preparation

First, 4–10-day-old male chickens were killed by CO_2_ inhalation and exsanguination. Chick biventer cervicis nerve-muscle preparations were then dissected and mounted in 5 mL organ baths on wire tissue holders under 1 g resting tension. Organ baths were maintained at 34 °C and filled with physiological salt solution of the following composition (mM): 118.4 NaCl, 4.7 KCl, 1.2 MgSO_4_, 1.2 KH_2_PO_4_, 2.5 CaCl_2_, 25 NaHCO_3_ and 11.1 glucose. Indirect twitches of the preparation were evoked by stimulating the motor nerve (0.1 Hz; 0.2 ms) at supramaximal voltage (10–20 V), using a stimulator. The addition of d-tubocurarine (10 μM), and subsequent abolishment of twitches, confirmed the selective stimulation of the nerve. Tissues were then washed with physiological salt solution until twitch responses to nerve stimulation were restored. Contractile responses of the tissues to exogenous acetylcholine (ACh; 1 mM for 30 s), carbachol (CCh; 20 μM for 60 s) and KCl (40 mM for 30 s) were obtained in the absence of nerve stimulation. Nerve stimulation was then recommenced for at least 30 min before the addition of the venom or antivenom. At the conclusion of each experiment, ACh, CCh and KCl were re-added as above. Twitch responses and responses to exogenous agonists were measured via a Grass FT03 force displacement transducer and recorded on a PowerLab system (ADInstruments Pty Ltd., Bella Vista, Australia). 

#### 5.3.1. Antivenom Prevention Studies

To examine the ability of antivenom to neutralise the venom, tissues were equilibrated with antivenom for 10 min before the venom was added. 

#### 5.3.2. Antivenom Reversal Studies

To determine the ability of antivenom to reverse the venom-induced neurotoxicity, antivenom was added at *t*_90_ (i.e., time at which the initial twitch height was inhibited by 90%). 

### 5.4. Data Analysis

Nerve-mediated twitch responses and responses to ACh (30 s), CCh (60 s) and KCl (30 s) were measured via a Grass FT03 force displacement transducer and recorded on PowerLab system (ADInstruments Pty Ltd., Bella Vista, Australia). Post-venom responses were expressed as a percentage of their initial responses. A one-way analysis of variance (ANOVA) was used to compare the effect on twitch height by different pre-treatments. Comparison of responses to exogenous agonists before and after pre-treatment was made using a Student’s paired *t* test. All ANOVAs were followed by a Bonferroni’s multiple comparison post hoc test. Data presented are in the form of mean ± standard error of the mean (SEM) of n experiments. All data and statistical analyses were performed using PRISM 9.2.0 (GraphPad Software, San Diego, CA, USA, 2016). For all statistical tests, *p* < 0.05 was considered statistically significant.

## Data Availability

Not applicable.
